# Polypoid pulmonary arteriovenous malformation causing hemothorax treated with thoracoscopic wedge resection

**DOI:** 10.1186/s40792-018-0428-1

**Published:** 2018-03-12

**Authors:** Haruhiko Shiiya, Yasuhiro Suzuki, Shigeo Yamazaki, Kichizo Kaga

**Affiliations:** 1 Department of Cardiovascular and Thoracic Surgery, Hokkaido University Graduate School of Medicine, Kita 15 Nishi 7, Kita-ku, Sapporo, Hokkaido 060-8638 Japan; 2 Department of Surgery, Keiyukai Sapporo Hospital, Kita 1, Hondori 14, Shiroishi-ku, Sapporo, Hokkaido 003-0027 Japan

**Keywords:** Pulmonary arteriovenous malformation, Video-assisted thoracic surgery, Wedge resection, Hereditary telangiectasia

## Abstract

**Background:**

Pulmonary arteriovenous malformations (PAVMs) can be associated with life-threatening complications such as paradoxical embolization, cerebral abscess, and hemothorax. Therefore, all adults with PAVMs should be offered treatment. Percutaneous transcatheter embolization is the first-line treatment, but 5–25% of cases require further treatment due to persistence after embolization. Recently, the role of minimally invasive thoracic surgery as a definitive treatment has been evaluated. We describe a case of a small peripheral PAVM causing hemothorax, which was safely treated with video-assisted thoracoscopic surgery (VATS). In our case, the PAVM appeared to protrude into the pleural cavity on chest computed tomography (CT), perhaps explaining why it led to a hemothorax.

**Case presentation:**

A 64-year-old man with a history of a brain abscess, for which he underwent surgery 6 months previously, developed a left-sided hemothorax. He had experienced recurrent epistaxis and received anticoagulation therapy for chronic atrial fibrillation. Chest CT after drainage revealed a solitary 15-mm nodule in the periphery of the left lower lobe, and identification of a feeding artery and draining vein on three-dimensional CT suggested that the node was a PAVM. The PAVM was adjacent to the diaphragm and multi-detector CT (MDCT) and three-dimensional CT (3DCT) showed that the nodule slightly displaced the diaphragm and protruded into the pleural cavity. After discussion in a multidisciplinary conference, it was decided that surgical treatment would be preferable to catheter embolization. The patient underwent VATS with three ports, the largest of which was 15 mm. The PAVM protruded from the peripheral lung like a polyp, and wedge resection was performed after simple adhesiolysis. There were no complications, and the patient is asymptomatic after 1-year of follow-up.

**Conclusions:**

As in the present case, PAVMs protruding into the pleural cavity can cause hemothorax, and surgical wedge resection of the involved lung as a definitive treatment is feasible and possibly safer than catheter embolization, particularly if the PAVM is localized close to the visceral pleura. Protrusion into the pleural cavity (polypoid appearance) was detected using MDCT and 3DCT preoperatively.

## Background

Pulmonary arteriovenous malformations (PAVMs) are abnormal connections between pulmonary arteries and veins. Approximately 70% of PAVMs are associated with hereditary hemorrhagic telangiectasia (HHT), and 15–50% of patients with HHT have PAVMs [1, 2]. While the usual presenting symptoms include cutaneous telangiectasias or epistaxis due to HHT, PAVMs can be associated with life-threatening complications such as dyspnea, paradoxical embolization, cerebral abscess, hemoptysis, and hemothorax, all of which require urgent treatment using a variety of therapeutic modalities [2]. Percutaneous transcatheter embolization is the first-line therapy, but 5–25% of cases require re-treatment due to the persistence of blood flow after embolization [3]. Recently, the role of minimally invasive thoracic surgery as a definitive treatment has been reconsidered [4].

We describe the case of a small peripheral PAVM associated with HHT and causing a hemothorax, which was safely treated with video-assisted thoracoscopic surgery (VATS). In our case, the PAVM protruded into the pleural cavity and was detected on chest computed tomography (CT) preoperatively.

## Case presentation

**Fig. 1:**
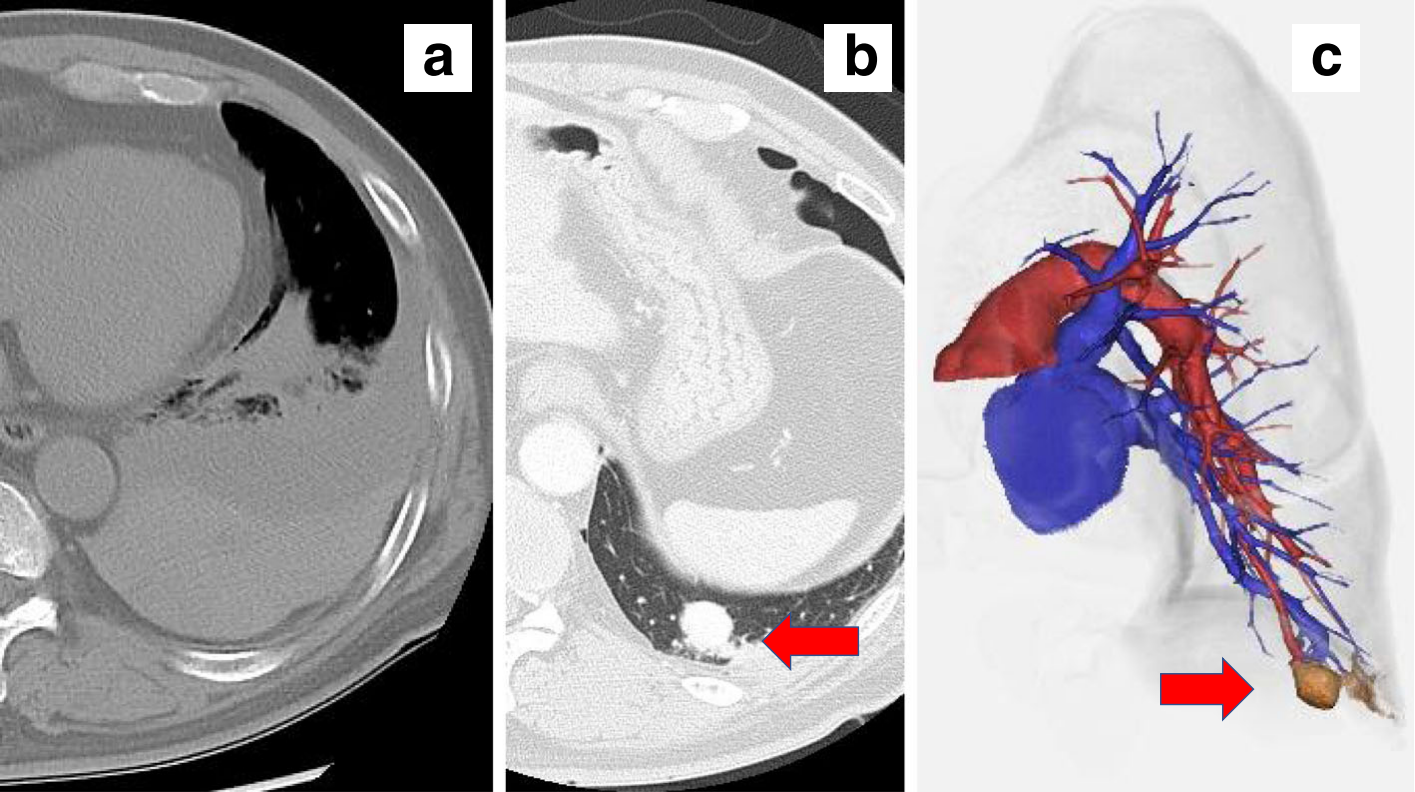
Chest computed tomography findings. **a** Chest CT showing left-sided hemothorax. **b** Chest CT after drainage showing a solitary nodule in the left lower lobe. **c** Three-dimensional CT showing the nodule to be suggestive of a PAVM

**Fig. 2:**
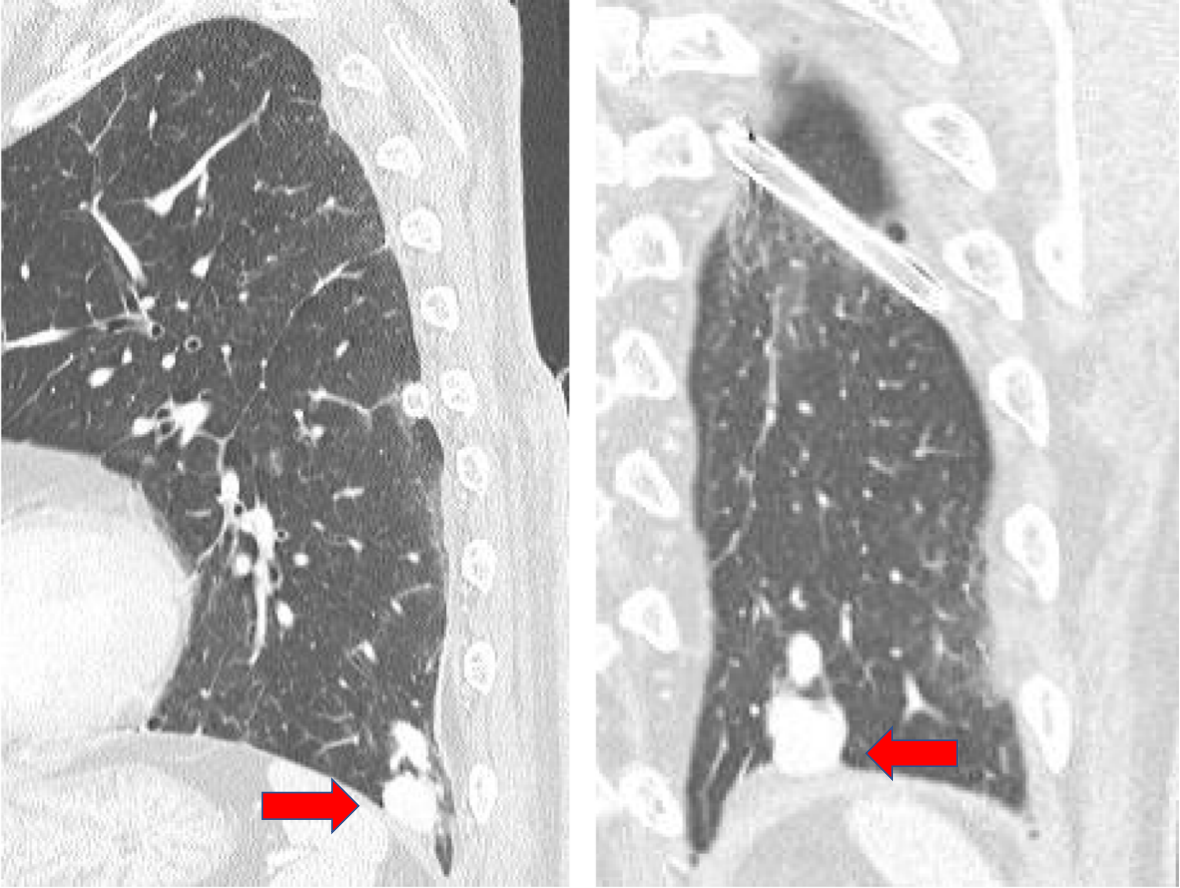
MDCT findings. The PAVM was adjacent to the diaphragm and slightly displaced the diaphragm, and protruded into the pleural cavity (red arrow)

A 64-year-old man was admitted to an outside hospital 6 months prior with a brain abscess and underwent surgery. He experienced recurrent epistaxis and was on anticoagulation therapy for chronic atrial fibrillation. During follow-up, he developed dyspnea, and chest CT showed a left-sided pleural effusion (Fig. 1a). Chest drainage revealed the collection to be a hemothorax, and the patient was referred to our hospital. Laboratory data showed slight anemia with a hemoglobin of 9.4 g/dl. Pulmonary function tests were within normal range. Chest CT after drainage revealed a solitary 15-mm nodule in the periphery of the left lower lobe, and identification of a feeding artery and draining vein on three-dimensional CT (3DCT) suggested that the node was a PAVM (Fig. 1b, c). The PAVM was adjacent to the diaphragm, and multi-detector CT (MDCT) and 3DCT demonstrated that the peripheral nodule slightly displaced the diaphragm, with the nodule protruding into the pleural cavity (Fig. 2). After multidisciplinary conference, we believed that surgical therapy might be preferable to catheter embolization for the following reasons. Firstly, the patient had already developed a brain abscess and hemothorax, so definitive treatment without a chance for recurrence was paramount. The second reason was that the PAVM was a solitary, small nodule located in the periphery of the lung, with MDCT and 3DCT indicating that the nodule might be in close proximity to the visceral pleura protruding into the pleural cavity, making minimally invasive wedge resection straightforward.

**Fig. 3:**
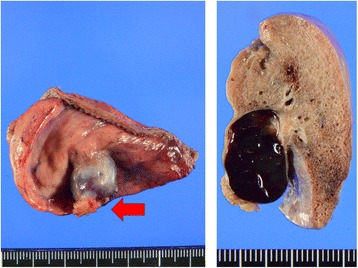
Resected left lower lobe specimen. The PAVM protruded into the pleural cavity. The ruptured region was covered by pericardial fat (red arrow)

The patient was placed in the right lateral decubitus position, and VATS with three ports was performed. The maximum incision length was 15 mm. The PAVM protruded from the periphery of the lung like a polyp, and wedge resection was easily performed after simple adhesiolysis (Fig. 3). There were no complications, and the patient remained asymptomatic after 1-year of follow-up.

## Discussion

In this report, we described a characteristic case of PAVM with a polypoid appearance (protrusion into the pleural cavity) in a patient with a history of a brain abscess. The patient presented with hemothorax, and the PAVM was easily and safely treated with VATS wedge resection. We performed VATS to treat the PAVM instead of electing for transcatheter embolization. The reasons for this were that, firstly, the PAVM was a solitary lesion located in the periphery of the lung; VATS wedge resection could thus be safely and easily undertaken. Secondly, as the patient had already experienced complications including a prior brain abscess and hemothorax, we sought to perform the safest and most definitive procedure. The PAVM protruded from visceral pleura into the pleural cavity, perhaps explaining why it ruptured.

In previous reports, the location and morphology of PAVMs have not always been described, so the frequency of PAVMs with a polypoid appearance as in our case is unclear. One review described that approximately 81% of PAVMs involved the pleura [5]. A case report [6] described subpleural PAVMs successfully treated with VATS, but the PAVMs were located in the interlobar surface of the right upper lobe, and there were multiple lesions. A new classification system may be needed to categorize PAVMs according to shape and location in order to help select the appropriate treatment.

In our case, the solitary PAVM was located in the left lower lobe and had a single feeding pulmonary artery and draining vein. According to some reviews, 53 to 70% of PAVMs are found in the lower lobes, 75% are unilateral, and 64% are solitary lesions. Eighty percent to 90% of PAVMs have a single feeding segmental artery and a single draining vein and are defined as simple type [1, 7]. PAVMs protruding into the pleural cavity are relatively rare, but a solitary PAVM located adjacent to the visceral pleura which can easily be treated with VATS wedge resection (as in our case) may be common. In rare cases, PAVMs have thrombosis in the nodule [1, 6]. Careful handling of the nodule is feasible to avoid iatrogenic thromboembolic diseases.

**Fig. 4:**
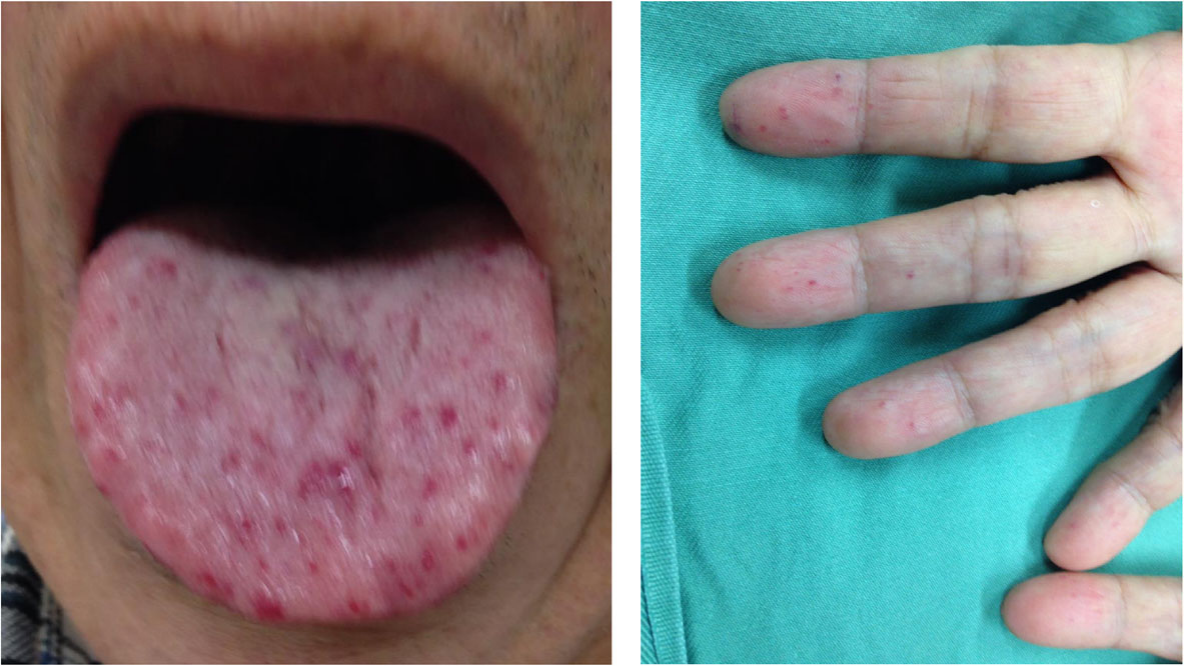
Physical findings. The patient had telangiectasias on his tongue and fingers

The diagnosis of HHT is based on clinical criteria or genetic testing. In our case, the patient was categorized as “definite” HHT with three of the four clinical criteria for diagnosis of HHT [2]. He experienced spontaneous and recurrent epistaxis, he had multiple telangiectasias (Fig. 4), and he had pulmonary arteriovenous malformations (visceral lesion). Family history was not available.

International guidelines for HHT suggest that there is no role for the surgical management of PAVMs other than in cases of life-threatening bleeding, except for cases wherein the patient is in a center where catheter embolization is unavailable [2]. However, the guidelines describe that reperfusion can occur in up to 15% of cases after embolization. Even though PAVM-related complications occurring after embolization are rare, closer follow-up may be needed, and the risk of paradoxical embolization is a possibility in those cases when reperfusion occurs. There are almost no PAVM-related complications after surgical resection when patients are appropriately selected. Although catheter embolization is a less invasive treatment than conventional surgery, the VATS procedure has also become less invasive as surgical instruments have progressed. Furthermore, there are few centers in Japan in which emergent catheter-based therapies are available. Surgical resection as a definitive therapy, therefore, should always be considered.

## Conclusions

PAVMs protruding into the pleural cavity can cause hemothorax, and surgical resection as a definitive treatment may be safer than catheter embolization and certainly may be more readily available from a resource standpoint. A solitary PAVM located adjacent to the visceral pleura can be removed easily and safely with VATS wedge resection with almost no risk of recurrence. Protrusion into the pleural cavity (creating a polypoid appearance) can be detected with MDCT and 3DCT preoperatively.

## Abbreviations

3DCT: Three-dimensional CT
CT: Computed tomography
HHT: Hereditary hemorrhagic telangiectasia
MDCT: Multi-detector CT
PAVMs: Pulmonary arteriovenous malformations
VATS: Video-assisted thoracoscopic surgery
